# Supplementary figures and images for: O-GlcNAcylation Affects the Pathway Choice of DNA Double-Strand Break Repair
Source: Int J Mol Sci. 2021 May 27;22(11):5715. doi: 10.3390/ijms22115715 (PMC8198441; doi:10.3390/ijms22115715)

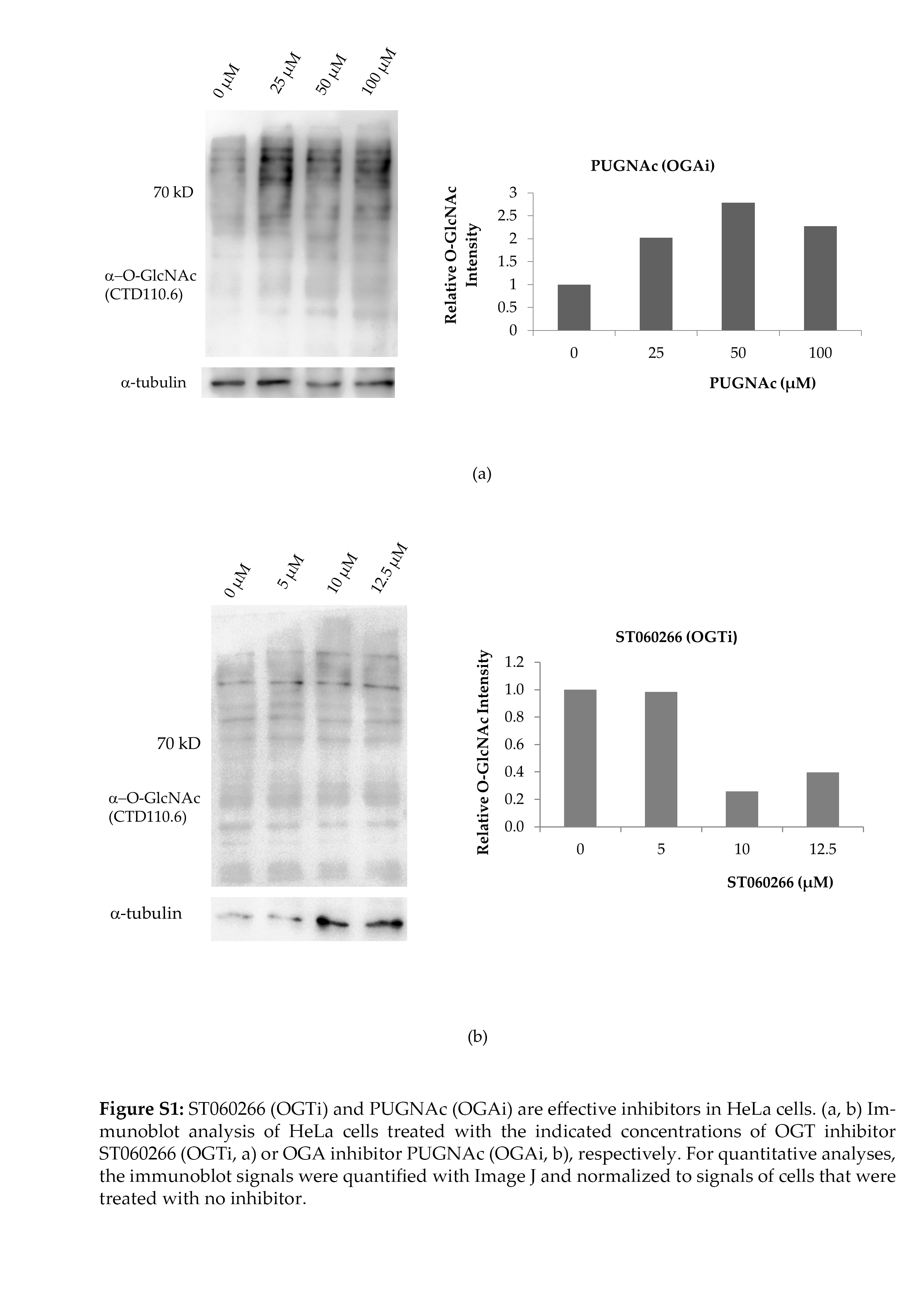

Supplement: Supplementary file 1 [file ijms-22-05715-s001.zip › Averbek_FigureS1_with caption_flattened_proofread.tif]

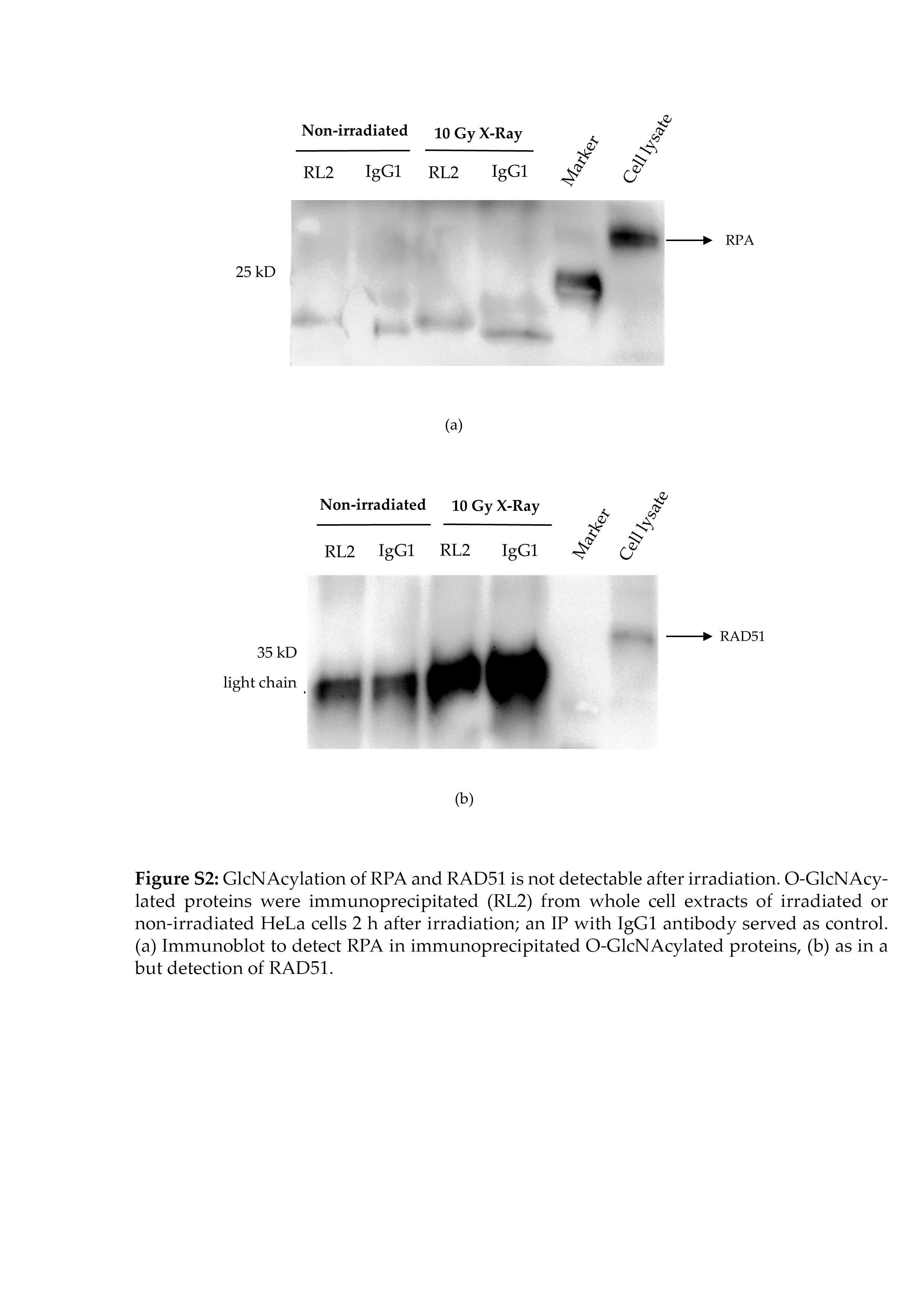

Supplement: Supplementary file 1 [file ijms-22-05715-s001.zip › Averbek_FigureS2_with caption_flattened_proofread.tif]
